# Fluctuations in prevalence of cervical human papillomavirus in women frequently sampled during a single menstrual cycle

**DOI:** 10.1038/sj.bjc.6600485

**Published:** 2002-08-12

**Authors:** M A P C van Ham, W J G Melchers, A G J M Hanselaar, R L M Bekkers, H Boonstra, L F A G Massuger

**Affiliations:** Department of Gynaecology and Obstetrics, University Medical Centre Nijmegen, PO Box 9101, 6500 HB, Nijmegen, The Netherlands; Department of Medical Microbiology, University Medical Centre Nijmegen, PO Box 9101, 6500 HB, Nijmegen, The Netherlands; Department of Pathology, University Medical Centre Nijmegen, PO Box 9101, 6500 HB, Nijmegen, The Netherlands

**Keywords:** human papillomavirus, menstrual cycle, prevalence

## Abstract

In the last few years much attention has been focused on the implementation of human papillomavirus detection in population based screening programmes to identify women at risk for cervical cancer. Short-term fluctuations in prevalence of human papillomavirus were investigated within a single menstrual cycle. The highest prevalence was found at the follicular phase (55%), whereas the cumulative prevalence was 75%.

*British Journal of Cancer* (2002) **87**, 373–376. doi:10.1038/sj.bjc.6600485 www.bjcancer.com

© 2002 Cancer Research UK

## 

Epidemiological and molecular studies over the past two decades have convincingly demonstrated that certain types of human papillomavirus (HPV) are etiologically related to the development of most cases of cervical cancer (Schiffman *et al*, 1993; Chichareon *et al*, 1998; Ngelangel *et al*, 1998; Liaw *et al*, 1999; Walboomers *et al*, 1999; Andersson *et al*, 2001; Lehtinen *et al*, 2001). A systematic review on the role of HPV testing within a cervical cancer screening program showed that the high risk HPV prevalence in CIN 1 ranged from 30 to 65%, in CIN 2 from 40 to 70% and in CIN 3 from 60 to 90% (Cuzick *et al*, 1999). Furthermore, HPV can be detected in almost all cervical carcinomas (Walboomers *et al*, 1999).

The estimated point prevalences of genital HPV infections detected by PCR based methods among populations of women with cytological normal cervical smears range from 1.5 to 44.3%, with a weighted average of 16.2% (Xi and Koutsky, 1997) and show an age-related pattern (Melkert *et al*, 1993). The highest prevalences are found in women in their early twenties (de Villiers *et al*, 1992; Melkert *et al*, 1993). Newly acquired genital HPV is usually transient in women with normal cervical cytology (Evander *et al*, 1995). The mean duration of cervical HPV infection in a healthy population varies between 8.2–13.5 months (Ho *et al*, 1998; Franco *et al*, 1999). Short-term fluctuations of an individual's HPV status, however, are still a matter of debate. Several studies have reported a fluctuating expression of HPV (Ho *et al*, 1998; Schneider *et al*, 1992; Wheeler *et al*, 1996), probably due to differences in viral load, inadequate sampling or reactivation of endogenous infection (Woodman *et al*, 2001). Single point measurements of HPV 16 in cervical smears are of limited value for assessment of an individual's HPV status (Schneider *et al*, 1992).

Before incorporation of HPV testing in screening programs for prevention of cervical cancer it is important to gain more insight in the actual prevalence of HPV, influenced by short-term fluctuations. The aim of this study was to investigate the presence of cervical HPV in the different phases of a single menstrual cycle in women with regular cycles.

## MATERIALS AND METHODS

Cervical samples for HPV detection and cytological examination were obtained from 20 women visiting the outpatient clinic for fertility problems at the University Medical Centre Nijmegen, the Netherlands. Only women with regular menstrual cycles were included. Patients with cervical treatment for abnormal cervical cytology in the past 10 years were not included in the study. After giving informed consent, patients were interviewed regarding their medical history, smoking habits, use of contraceptives and sexual behaviour.

Cervical smears for HPV detection and cytological classification were obtained using the Cervex brush (Rovers, Oss, the Netherlands).

On a weekly basis at four visits during their fertility screening cycle, cervical swabs were taken for HPV detection. The first cervical sample was taken in the menstrual period (2nd or 3rd day of the cycle), the second sample between the 7th and 11th day of the cycle, the third sample around ovulation (between the 12th and 15th day) and the fourth sample in the luteal phase (7 days post ovulatory, 20th to 24th day). Ovulation was ascertained by ultrasound examination. Cytological classification was done by an experienced pathologist.

Cervical scrapes were processed into AgarCyto cellblocks as described by Kerstens *et al* (2000). HPV detection was assessed using a short fragment polymerase chain reaction (SPF_10_-PCR). HPV genotyping was performed via a reverse hybridisation line probe assay (LiPA), capable of detecting and genotyping 25 different HPV types simultaneously (Kleter *et al*, 1998, 1999). Statistical analysis was performed using the chi-square and McNemar tests, where appropriate. Values of *P*<0.05 were considered to indicate a significant difference in HPV prevalence between different phases in the menstrual cycle.

## RESULTS

Eighty cervical smears for HPV testing were obtained from 20 women with a mean age of 33.8±s.d. 4.9 years (range 22–43 years). All women except one were non-smokers. The use of oral contraceptives was discontinued by all, at least 1 year before attending our fertility clinic. The mean age of sexarche was 18.6±4.5 years. Nine women had been monogamous since their sexarche, while the other 11 women were at least monogamous for the past 2 years. The mean frequency of sexual intercourse was 2.3 weekly.

Chlamydia serum antibodies were present in four patients. Urine samples from these four women, obtained to test for an active chlamydia infection, were all negative.

Three patients (15%) had a history of abnormal cervical smears. Two of them had ASCUS in 1986 (Table 1

**Table 1:**
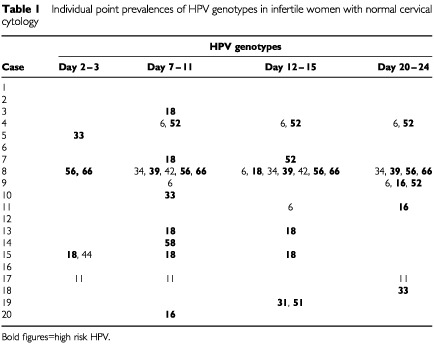
Individual point prevalences of HPV genotypes in infertile women with normal cervical cytology

, case no. 12, HPV negative) and in 1996 (Table 1, case no. 10, HPV 33 positive) respectively. The third woman (Table 1, case no. 6, HPV negative) was treated in 1984 by a large loop excision of the transformation zone, which was histologically classified as severe dysplasia. The follow-up smears of this patient were normal.

**Figure 1:**
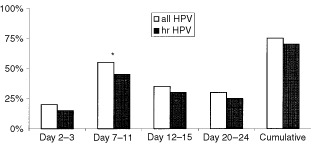
Point-prevalences and cumulative prevalence of HPV during one menstrual cycle in infertile women. *P<0.05 hr=high risk.

At the end of the study 15 (75%) women appeared to be positive for HPV at least one time within one menstrual cycle. Single point measurements of HPV ranged from 20 to 55% (Figure 1

). The cumulative prevalence of oncogenic HPV was 70% (14 out of 20), with point-prevalences fluctuating between 15 and 45% (Figure 1).

In the follicular phase (7th to 11th day) a higher rate of HPV positive samples was found compared to the other phases, which was significantly different according to the chi-square test (*P*=0.02).

Fifteen different HPV genotypes were detected of which 73.3% were high risk. Three (15%) patients harboured HPV 16 in their lower genital tract, whereas five (25%) women tested positive for HPV 18 for at least one time within the menstrual cycle (Table 1). Chlamydia serum antibodies were found positive in three women with HPV 18 in their cervices. Interestingly, HPV 18 was only detected in the first half of the menstrual cycle and never in the luteal phase.

Five women in the HPV positive group (33.3%) contained multiple HPV genotypes in one or more of their cervical smears. In one patient seven different oncogenic HPVs were detected of which type 56 and 66 were consistently present (Table 1, case no. 8). She was the youngest participant (22 years) and had her first sexual experience at the age of 14 years. She was monogamous for the last 2.5 years, but counted at least 10 different sexual partners in the past. The cervical smear of this patient was cytological classified as ASCUS.

The only other patient with ASCUS (Table 1, case no. 11) was positive for HPV 16 in the luteal phase. The cervical smears of the other 18 women were cytologically diagnosed as normal.

## DISCUSSION

In this study, the cumulative prevalence of HPV in infertile, cytologically normal women following weekly consecutive cervical scrapes during one menstrual cycle was found to be 75%. To our knowledge this is the highest cumulative prevalence that has ever been reported for women with normal cervical cytology after such a short follow-up of only 4 weeks. Several other studies demonstrated cumulative cervical HPV prevalence in cytologically normal women of 34.7 to 66.7% (de Villiers *et al*, 1992; Schneider *et al*, 1992; Wheeler *et al*, 1996; Ho *et al*, 1998; Woodman *et al*, 2001). However, in those studies multiple samples were taken during study periods of 10 weeks to 5 years in contrast to 4 weeks in our study (Table 2

**Table 2:**
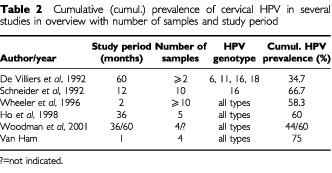
Cumulative (cumul.) prevalence of cervical HPV in several studies in overview with number of samples and study period

). Moreover, we studied the prevalence of all HPV genotypes in contrast to Schneider (only HPV 16) and de Villiers (HPV 6, 11, 16, 18). In a group of Panama City prostitutes cumulative HPV prevalence amounted 82% after 8 months with at least six repeated examinations (Reeves *et al*, 1989). However, cytological classification of these women was not reported.

In our study, point prevalence of HPV genotypes changed throughout the menstrual cycle with the highest prevalence in the follicular phase. From then onwards, single-point HPV prevalence decreases until the end of menstruation. Exfoliation of cervical cells by scraping the surface of the cervix consecutively every week could have led to a reduction in cells containing HPV, resulting in a lower prevalence of HPV with every following smear. However, the initial smears have been made at the menstrual period at which the lowest prevalence of HPV was found.

Single time HPV detection rates in our study varied between 20 and 55%, which is comparable to earlier reports (Schneider *et al*, 1992; Wheeler *et al*, 1996). It has been postulated that variations of the single time prevalence are due to differences in viral load. Viruses can be persistent but undiagnosed because fluctuating levels of infection around the threshold of detectability (Woodman *et al*, 2001).

We found a significantly higher prevalence of HPV in the follicular phase, with predominance of HPV 18 (38.4%). Whereas others found a significantly higher prevalence of HPV in the luteal phase of the cycle (Schneider *et al*, 1992). However, in this last study, women were only tested for HPV genotype 16. Wheeler *et al* (1996) reported no significant correlation in HPV detection and phase of menstrual cycle, although the point prevalence of HPV in the follicular phase was higher than in the luteal phase. Fairley *et al* (1994) performed a study to determine if HPV detection or the size of the tampon specimen was effected by the menstrual cycle. They concluded from their study that the timing of the menstrual cycle effects the size of tampon specimens but not the probability of detecting HPV DNA.

The high prevalence of HPV in the follicular phase of our study group is remarkable. A possible explanation for this finding is the physiological widening of the cervical canal in the follicular phase, resulting in the collection of more or different clinical material from the deeper layers of the endocervix. This could also be an explanation for the predominance of HPV 18 in the follicular phase. After all, in a recent study of the prevalence of HPV in cervical adenocarcinomas it was demonstrated that HPV 18 was the most prevalent followed by HPV 16 (Andersson *et al*, 2001).

It could be argued that fertility problems *per se* could have led to a population based bias in HPV prevalence. However, the cervical HPV prevalence in a group of infertile women undergoing ovarian stimulation with gonadotrophins varied between 6.8 and 7.8 %, which was similar to 8.4% in the control group (Strehler *et al*, 1999).

In women with normal cytological cervical smears, a cumulative prevalence of HPV as high as 75% in one menstrual cycle has never been reported. This result suggests that single point detection of HPV underestimates the true prevalence of HPV in a population. Sequential scrapes appear to be necessary to determine the true HPV status in individual cases.

We conclude from our study that detection of HPV on single sample basis may lead to false negative results. These results may have important implications for population based HPV screening.
